# *Sulfurimonas microaerophilic* sp. nov. and *Sulfurimonas diazotrophicus* sp. nov.: Two Novel Nitrogen-Fixing and Hydrogen- and Sulfur-Oxidizing Chemolithoautotrophs Within the *Campylobacteria* Isolated from Mangrove Sediments

**DOI:** 10.3390/microorganisms13040713

**Published:** 2025-03-21

**Authors:** Yangsheng Zhong, Yufei Li, Zhaodi Wang, Liang Cui, Shiwei Lv, Han Zhu, Qing Yuan, Qiliang Lai, Shasha Wang, Lijing Jiang

**Affiliations:** 1 Key Laboratory of Marine Genetic Resources, Third Institute of Oceanography, Ministry of Natural Resources of PR China, Xiamen 361005, China; zys6411@163.com (Y.Z.); dittoli0703@163.com (Y.L.); wzd11080213@163.com (Z.W.); cuiliang@tio.org.cn (L.C.); lsw13105292223@163.com (S.L.); 2021210128@jou.edu.cn (Q.Y.); laiqiliang@tio.org.cn (Q.L.); 2 Fujian Key Laboratory of Marine Genetic Resources, Xiamen 361005, China; 3 School of Marine Biology, Xiamen Ocean Vocational College, Xiamen 361012, China

**Keywords:** *Sulfurimonas microaerophilic* HSL1-7^T^, *Sulfurimonas diazotrophicus* HSL3-1^T^, chemolithoautotroph, hydrogen oxidation, sulfur oxidation, nitrogen fixation

## Abstract

Two novel marine hydrogen- and sulfur-oxidizing bacteria, designated HSL1-7^T^ and HSL3-1^T^, were isolated from mangrove sediments from Fujian Province, China. Strain HSL1-7^T^ exhibited Gram-negative, rod-shaped to slightly curved morphology with polar flagellum-driven motility, whereas strain HSL3-1^T^ was Gram-negative, rod-shaped and non-motile. Strain HSL1-7^T^ and HSL3-1^T^ were obligate chemolithoautotrophs, capable of using molecular hydrogen and thiosulfate as an energy source, and molecular oxygen and elemental sulfur as the electron acceptors for growth. Cellular fatty acid profiles revealed similar predominant components (C_16:1_*ω7c*, C_16:0_, C_18:1_*ω7c*, and C_14:0_) in both strains. Strains HSL1-7^T^ and HSL3-1^T^ were strongly diazotrophic, as demonstrated by ^15^N_2_ fixation when a fixed nitrogen source was absent from the growth medium. The DNA G+C contents of strains HSL1-7^T^ and HSL3-1^T^ were determined to be 36.1% and 57.3%, respectively. Based on the 16S rRNA gene sequences, strains HSL1-7^T^ and HSL3-1^T^ exhibited the highest sequence similarities with *Sulfurimonas marina* B2^T^ (98.5% and 94.45%, respectively). Notably, the 16S rRNA gene sequence similarity between strains HSL1-7^T^ and HSL3-1^T^ was 93.19%, indicating that they represent distinct species within the genus *Sulfurimonas*. Comparative genomic analyses revealed the presence of diverse metabolic profiles in strains HSL1-7^T^ and HSL3-1^T^, including carbon fixation, hydrogen oxidation, sulfur oxidation, and nitrogen fixation. The combined phenotypic, chemotaxonomic, and phylogenetic evidence, including average nucleotide identity and in silico DNA-DNA hybridization values, shows that strains HSL1-7^T^ and HSL3-1^T^ represent two novel species of the genus *Sulfurimonas* for which the names *Sulfurimonas microaerophilic* sp. nov. and *Sulfurimonas diazotrophicus* sp. nov. are proposed, with the type strains HSL1-7^T^ (=MCCC 1A18899^T^ = KCTC 25640^T^) and HSL3-1^T^ (=MCCC 1A18844^T^), respectively.

## 1. Introduction

Mangrove sediments are typically recognized as organic-rich but nitrogen-limited ecosystems [1,2]. As the rate-limiting step of nitrogen cycling, nitrogen fixation is particularly important to alleviate the nitrogen limitation of mangrove ecosystems. Our recent studies have demonstrated that chemolithoautotrophs, such as members of *Campylobacterota*, *Proteobacteria*, and *Nitrospirota,* are the dominant nitrogen fixers in mangrove sediments, rather than heterotrophs [3]. This study underscores the significance of chemolithoautotrophs in carbon-dominant ecosystems. Compared with heterotrophic nitrogen-fixing bacteria [4], fewer chemolithoautotrophic diazotrophs have been cultured so far. Therefore, isolating new related strains is conducive to deepening our understanding of the dark nitrogen fixation of this group in mangrove sediments.

The genus *Sulfurimonas,* belonging to the class *Campylobacteria* (formerly *Epsilonproteobacteria*) within the phylum of *Campylobacterota* (formerly *Proteobacteria*), was first proposed by Inagaki et al. in 2003 [5,6,7]. Members of the genus *Sulfurimonas* are globally distributed in sulfidic environments, including coastal sediments, pelagic redoxclines, deep-sea hydrothermal vents, and terrestrial soils [7,8]. They play an important role in the marine carbon, nitrogen, and sulfur cycles. The most widely shared feature of species of the genus *Sulfurimonas* is chemolithoautotrophic growth. A variety of electron donors and acceptors, such as molecular hydrogen (H_2_) and reduced sulfur compounds, are commonly utilized as the electron donors, and molecular oxygen (O_2_), nitrate (NO_3_^−^), and elemental sulfur as the common electron acceptors. This may contribute to their widespread distribution [9,10,11]. The unique feature of sulfur-oxidizing coupled denitrification enables its application in wastewater denitrification [12], thereby facilitating the development of novel environmental remediation technologies.

At the time of writing, the genus *Sulfurimonas* comprises ten species with validly published names and three Candidatus species; these can be found in an up-to-date website (https://lpsn.dsmz.de/genus/sulfurimonas accessed on 1 November 2024). Among these, five species were isolated from deep-sea hydrothermal vents environments, including *Sulfurimonas indica* NW8N^T^, *Sulfurimonas autotrophica* OK10^T^, *Sulfurimonas hydrogeniphila* NW10^T^, *Sulfurimonas paralvinellae* GO25^T^, and *Sulfurimonas sediminis* S2-6^T^ [7,13,14,15,16]. Four species were isolated from coastal marine sediments, including *Sulfurimonas xiamenensis* 1-1N^T^, *Sulfurimonas denitrificans* DSM 1251^T^, *Sulfurimonas hongkongensis* AST-10^T^, and *Sulfurimonas lithotrophica* GYSZ_1^T^ [17,18,19]. *Sulfurimonas marina* B2^T^ was isolated from a deep-sea sediment sample. *Sulfurimonas crateris* SN118^T^ was isolated from a terrestrial mud volcano, and *Sulfurimonas aquatica* H1576^T^ was isolated from a brackish lake, while *Sulfurimonas gotlandica* GD1^T^, *Candidatus Sulfurimonas baltica* GD2, and *Candidatus Sulfurimonas marisnigri* SoZ1 were isolated from the pelagic redoxcline of the Baltic Sea and Black Sea [20,21,22,23,24].

In this study, we report two novel strains, designated HSL1-7^T^ and HSL3-1^T^, isolated from mangrove sediments sampled at the Jiulong River estuary, Fujian Province, China. Their physiological properties and an 16S rRNA gene sequence analysis indicate that strains HSL1-7^T^ and HSL3-1^T^ represent members of the genus *Sulfurimonas*. A comparative genomic analyses indicates that they exhibit diverse metabolic profiles, especially their unique nitrogen fixation capacity, which enables us to further elucidate their potential ecological functions within mangrove ecosystems.

## 2. Materials and Methods

### 2.1. Isolation of the Novel Strain and Cultivation

Sediment samples were collected from Zhangzhou mangroves in the Jiulong River estuary, Fujian Province, China (24°23’33” N, 117°54’11” E), and transported to the laboratory at 4 °C. For microbial enrichment, approximately 1 g of fresh sediment was aseptically inoculated into the MMJHS medium, followed by incubation at 28 °C for 7 days under a gas mixture of 76% H_2_/20% CO_2_/4% O_2_ (200 kPa) according to established protocols [25]. The MMJHS medium consisted of NaCl (30 g l^−1^), NH_4_Cl (0.25 g l^−1^), KCl (0.33 g l^−1^), CaCl_2_·2H_2_O (0.14 g l^−1^), MgCl_2_·6H_2_O (4.18 g l^−1^), K_2_HPO_4_ (0.14 g l^−1^), NaHCO_3_ (1 g l^−1^), Na_2_S_2_O_3_·5H_2_O (10 mM), Wolfe’s vitamins (1 mL l^−1^), and a trace element solution (10 mL l^−1^) [26]. After successful enrichment with the MMJHS medium, strains HSL1-7^T^ and HSL3-1^T^ were successfully isolated as axenic cultures using the dilution-to-extinction method [27]. The purity of the cultures was confirmed by microscopic examination coupled with a full-length 16S rRNA gene sequence analysis.

### 2.2. Phenotypic and Chemotaxonomic Analyses

Gram staining was performed following the manufacture’s protocol (Hangzhou Tianhe Microorganism Reagent, Hangzhou, China) using the standard Schaeffer-Fulton method with quality control strains included for validation. The cell morphology was observed using the cells grown in the MMJHS liquid medium at 32 °C for one day, using a transmission electron microscopy (TEM, JEM-1230, JEOL, Tokyo, Japan). The growth of strains HSL1-7^T^ and HSL3-1^T^ was measured via direct cell counting under a phase-contrast microscope using a hemocytometer. To determine the physiological characteristics of both strains, triplicate cultures grown in the MMJHS medium were analyzed under each experimental condition.

The growth temperature was determined by incubating the cultures in the MMJHS medium for 24 h across a temperature gradient from 4 to 55 °C (4, 10, 15, 20, 25, 28, 30, 35, 37, 40, 45, 50, and 55 °C). The salinity tolerance was assessed using NaCl concentrations ranging from 0 to 9.0% (*w*/*v*) in 0.5% increments. For pH profiling, cultures in the MMJHS medium were incubated for 24 h across pH 4.0–9.0 (0.5 pH unit intervals) with specific buffer systems: acetate/acetic acid (pH 4.0–5.0), MES (pH 5.0–6.0), PIPES (pH 6.0–7.0), HEPES (pH 7.0–7.5), and Tris/CAPSO (pH 8.0 and above) [28]. Oxygen requirements were evaluated under controlled atmospheres with O_2_ concentrations from 0 to 20% (0, 1, 2, 4, 6, 8, 10, 12, 15, and 20% at 200 kPa). In the case of oxygen absence, 10 mM nitrate was added as a potential electron acceptor.

The utilization of alternative electron donors was evaluated by substituting sulfite (5 mM), thiocyanate (5 mM), tetrathionate (5 mM), elemental sulfur (1% *w*/*v*), or sodium sulfide (50–100 μM), for thiosulfate in the MMJS medium with a gas phase of 76% N_2_/20% CO_2_/4% O_2_ (200 kPa). The molecular hydrogen was also examined in the MMJH medium in the absence of thiosulfate under a gas phase of 76% H_2_/20% CO_2_/4% O_2_ (200 kPa). For the electron acceptor analysis, nitrate (10 mM), nitrite (5 mM), ferric citrate (20 mM), ferrihydrite (20 mM), and manganese (IV) oxide (200 μM) were tested with thiosulfate as the sole electron donor under 80% N_2_/20% CO_2_ (200 kPa).

The metabolic capability of inorganic nitrogen sources was evaluated by supplementing the nitrogen-deprived MMJHS medium (lacking all nitrogen sources) with ammonium chloride (5 mM), sodium nitrate (5 mM), or sodium nitrite (5 mM). Cultures were incubated under a gas phase of 76% H_2_/20% CO_2_/4% O_2_ (200 kPa). Utilization of N_2_ was also tested under a 76% N_2_/20% CO_2_/4% O_2_ (200 kPa) gas phase. Potential nutrients required for growth, such as selenite, tungstate, and vitamins, were examined with the MMJHS medium with, and without, the specified nutrients.

The heterotrophic growth capability was investigated in the MMJHS medium by substituting NaHCO_3_ with various organic carbon sources under a gas phase of 96% N_2_/4% O_2_ (200 kPa). To determine whether there was culture after 24 h of incubation, direct cell counting using a hemocytometer was carried out. These organic carbon sources included: 0.1% (*w*/*v*) peptone, starch, tryptone, casein, yeast extract, and casamino acids; 5 mM acetate, citrate, formate, succinate, propionate, tartrate and pyruvate; 5 mM of each of 20 amino acids; and 0.02% (*w*/*v*) sucrose, galactose, glucose, fructose, lactose, maltose, and trehalose. To systematically identify the alternative energy sources, these organic compounds were used as an energy source in the MMHJS medium to replace thiosulfate under a gas phase of 76% N_2_/20% CO_2_/4% O_2_ (200 kPa).

The whole-cell fatty acid profiles were analyzed using the MIDI Sherlock Microbial Identification System (v 6.0B). Cultures grown in the MMJHS medium (32 °C, 24 h) underwent sequential saponification, extraction, and methylation. Fatty acid methyl esters (FAMEs) were separated via an Agilent 6850 gas chromatograph and identified against the TSBA 6.0 reference database [29].

### 2.3. Phylogeny Analysis Based on the 16S rRNA Gene Sequences

The genomic DNA isolation from strains HSL1-7^T^ and HSL3-1^T^ was performed with a commercial bacterial DNA extraction kit (SBS Genetech Co., Ltd., Shanghai, China) following the manufacturer’s protocol. The DNA purity and concentrations were determined using a NanoDrop 2000 spectrophotometer (Thermo Scientific, Waltham, MA, USA). The amplification of the full-length 16S rRNA genes was achieved with the universal primer pair 27F (5′-AGAGTTTGATCMTGGCTCAG-3′) and 1492R (5′-GGTTACCTTGTTACGACTT-3′), as previously described [30]. The 16S rRNA genes were sequenced via the Sanger method (Applied Biosystems^TM^ 3730XL, Waltham, MA, USA) by Sangon Biotech (Shanghai) Co., Ltd., Shanghai, China. The full-length 16S rRNA gene sequencing was performed using the EzTaxon-e server [31]. The sequences of related taxa were obtained from the GenBank database. The phylogenetic reconstruction was conducted in MEGA 7.0 [32] employing three distinct algorithmic approaches: neighbor-joining [33], maximum-likelihood [34], and minimum evolution [35]. The multiple alignment was first performed using CLUSTAL_W, followed by an evolutionary distance calculation through Kimura’s two-parameter model [36]; the bootstrap values were determined based on 1000 replications [37].

### 2.4. Genome Analysis and Functional Annotation

The complete genome of strains HSL1-7^T^ and HSL3-1^T^ was sequenced by Shanghai Majorbio Bio-Pharm Technology Co., Ltd. (Shanghai, China) on a Pacific Biosciences (PacBio, Menlo Park, CA, USA) sequencing platform using a single-molecule real-time (SMRT) technology. The sequenced reads were filtered and the high-quality paired-end reads were assembled to reconstruct the circulating genome using SOAPdenovo2 software (v 2.0) [38]. The genomic DNA G+C content was determined from the complete genome sequences. To identify tRNA and rRNA within the genome, tRNAscan-SE and rRNAmmer were utilized [39]. The complete 16S rRNA gene sequence was extracted from the whole genome using RNAmmer [40]. The gene prediction was performed using Glimmer (v 3.02) [41]. The genome annotation was carried out using RAST server [42], METABOLIC (v 4.0) [43] and KofamtKOALA [44] against the KEGG database. An orthoANI algorithm [45] was implemented to calculate the average nucleotide identity (ANI) values between the paired genomes. The digital DNA-DNA hybridization (dDDH) values were estimated through the Genome-to-Genome Distance Calculator (GGDC) platform (https://ggdc.dsmz.de/, accessed on 5 December 2024). The phylogenomic tree was reconstructed with the Up-to-date Bacterial Core Gene (UBCG) pipeline (v 3.0) [46]. The phylogenomic tree was visualized and decorated using ChiPlot (https://www.chiplot.online/, accessed on 25 December 2024) [47].

### 2.5. Characterization of N_2_ Fixation

The nitrogen-fixing ability of isolates in this study were determined using the ^15^N_2_ assimilation method, as previously described [3]. Cells were grown microaerobically in a 60 mL serum bottle containing 10 mL of the nitrogen-free MMJHS medium, under a gas phase of 70% H_2_/20% CO_2_/4% O_2_ (200 kPa). 10 mL of ^15^N_2_ (99% atomic ^15^N; Isotec) was added to the growth medium. The nitrogen fixation ability was also tested by the addition of inorganic nitrogen compounds including 1 mM or 40 mM NH_4_Cl. In addition, the strain *Sulfurimonas xiamenensis* 1-1N^T^, isolated from the coastal sediments of Xiamen Island, Fujian Province, China, was used as the control; this lacked nitrogen fixation gene clusters. All the cultures were incubated at 32 °C in the dark for 24 h, and subsequently harvested by centrifugation (10,000× *g*, 4 °C, 20 min) when visible turbidity was observed. The cells were then washed twice with ice-cold 20 mM Tris buffer prepared in artificial seawater, followed by freeze-drying overnight [48]. The ^15^N atomic percentage of freeze-dried cells was determined by a CarloErba elemental analyzer (Model NA 1500, Fisons Instruments, San Carlos, CA, USA) couple with a Finnegan MAT (ThermoQuest, Temecula, CA, USA) Delta S isotope ratio mass spectrometer [49].

## 3. Results and Discussion

### 3.1. Morphological and Phenotypic Characteristics

The cells of the strain HSL1-7^T^ were Gram-stain-negative, rods to slightly curved, with dimensions of approximately 1.8–2.3 μm in length and 0.4–0.6 μm in width and exhibited motility via a single polar flagellum (Figure S1a). The cells of strain HSL3-1^T^ were Gram-stain-negative, rod-shaped, with dimensions of approximately 0.7–2.5 μm in length and 0.2–0.5 μm in width, and non-motile with no flagellum observed in the MMJHS medium (Figure S1b). Strain HSL1-7^T^ exhibited growth over a temperature range of 4 °C to 40 °C (optimum 32 °C). It also grew in NaCl concentrations from 1.0 to 4.0% (*w*/*v*) (optimum 3.0%). The pH range for growth was 5.0 to 8.5 (optimum pH 7.0). Additionally, growth was observed in oxygen concentrations ranging from 1% to 10% (optimum 4% O_2_). No growth was observed without oxygen, or with over 15% in the headspace gas (Table 1). Growth of strain HSL3-1^T^ was observed at temperatures ranging from 4 °C to 45 °C (optimum 37 °C), at NaCl concentrations ranging from 2.0–4.0% (*w*/*v*) (optimum 3.0%), at a pH range of 5.0–8.5 (optimum pH 7.0), and at an O_2_ range of 0–20% (optimum 6% O_2_). Strains HSL1-7^T^ and HSL3-1^T^ both exhibit morphological and phenotypic similarities with the other species in the *Sulfurimonas* genus, including growth temperature range, pH range, and maximum O_2_ concentration. However, there were some differential features between strains HSL1-7^T^ and HSL3-1^T^ and the other reference strains, as shown in Table 1.

Physiological characterization through the chemoautotrophic cultivation experiments revealed that strains HSL1-7^T^ and HSL3-1^T^ could grow with molecular hydrogen and thiosulfate as the energy sources, while utilizing molecular oxygen and elemental sulfur as electron acceptors. Nitrate and nitrite could not be utilized as electron acceptors. The results of the inorganic nitrogen source tests indicated that strains HSL1-7^T^ and HSL3-1^T^ could grow with ammonium and molecular nitrogen as the nitrogen source. Nitrite, tungsten, selenium, and vitamin supplementation were not required for growth. The results of the heterotrophic growth tests indicated that none of the organic compounds sustained the growth of these two strains as the carbon or energy source. These results indicate that strains HSL1-7^T^ and HSL3-1^T^ are obligate chemolithoautotrophs.

### 3.2. Chemotaxonomy

To analyze the cellular fatty acid, strains HSL1-7^T^ and HSL3-1^T^, and the closely related species, were cultivated in the MMJHS medium at 32 °C for 24 h. The fatty acid compositions of strains HSL1-7^T^ and HSL3-1^T^ and the closely related type species of the genus *Sulfurimonas* are listed in Table 2. The major cellular fatty acids of strains HSL1-7^T^ and HSL3-1^T^ were C_16:1_*ω7c*, C_16:0_, C_18:1_*ω7c*, and C_14:0_, which are similar to those of *S. marina* B2^T^, *S. lithotrophica* GYSZ_1^T^, and *S. xiamenensis* 1-1N^T^. However, differences were observed in the relative abundance of some fatty acids between strains HSL1-7^T^ and *S. marina* B2^T^. For example, the fatty acid C_16:1_*ω5c* was observed at 0.6% in HSL1-7^T^ but was not found in *S. marina* B2^T^. The fatty acid C_16:1_*ω7c* was observed at 28.7% in HSL1-7^T^, while it was observed at 37.7% in *S. marina* B2^T^. Some differences in fatty acids profiles were also observed between strains HSL3-1^T^ and *S. marina* B2^T^; the fatty acid C_12:0_ was observed at 4.0% in HSL3-1^T^, while it was only observed at 1.3% in *S. marina* B2^T^. The fatty acid C_16:1_*ω5c* was observed at 0.4% in HSL3-1^T^ but not found in *S. marina* B2^T^ (Table 2). In addition, there were some differences between strains HSL1-7^T^ and HSL3-1^T^. The fatty acid C_12:0_ was observed at 0.3% in HSL1-7^T^, while it was observed at 4.0% in HSL3-1^T^. The fatty acid of C_18:0_ was observed at 2.6% in HSL3-1^T^ but was observed at 0.7% in HSL1-7^T^ (Table 2).

### 3.3. Phylogeny of 16S rRNA Gene Sequences

**Figure 1:**
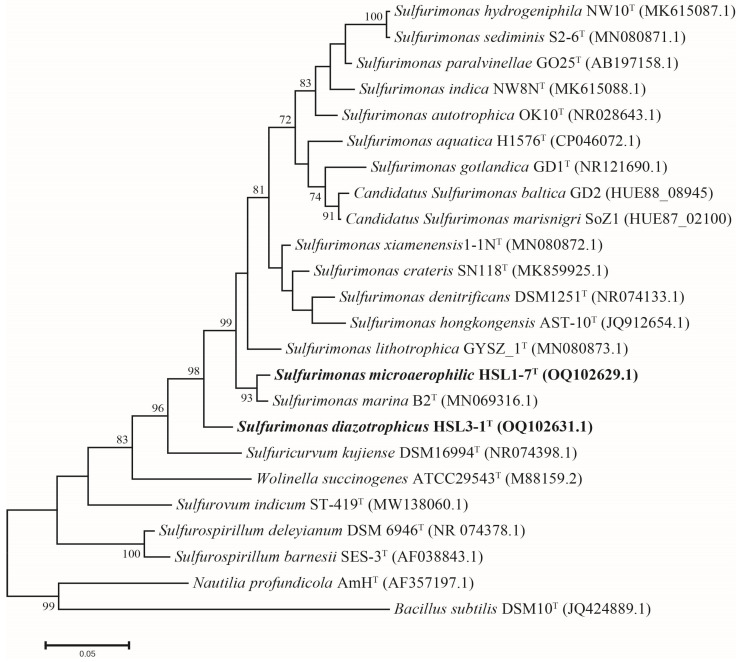
Maximum-likelihood phylogenetic tree based on the 16S rRNA gene sequences showing the relationship between strains HSL1-7^T^ and HSL3-1^T^ with other members within the genus *Sulfurimonas*. Species names highlighted in bold represent novel taxa discovered in this work. Bootstrap numbers (>70%) were shown with 1000 calculations. Bar, 0.05 substitutions per nucleotide position.

The comparative analysis of the full-length 16S rRNA gene sequences (1450bp) revealed that strain HSL1-7^T^ shares the closest phylogenetic affiliation with *S. marina* B2^T^, exhibiting a 98.45% sequence identity; the other strains shared sequence similarities below 95.5%. Strain HSL3-1^T^ was also most closely related to *S. marina* B2^T^ with a 94.45% sequence identity; the other strains shared sequence similarities below 94.0%. These sequence similarities were lower than the threshold of 98.65% for species delineation [50]. The phylogenetic analysis using the maximum-likelihood method revealed that strain HSL1-7^T^ was clustered with *S. marina* B2^T^, and strain HSL3-1^T^ was clustered with the other reference strains of *Sulfurimonas* (Figure 1). The phylogenetic topology was further supported by neighbor-joining and minimum-evolution analyses, which indicates that strains HSL1-7^T^ and HSL3-1^T^ represent members of the genus *Sulfurimonas* (Figures S2 and S3).

### 3.4. Genomic Properties

The genome size of strains HSL1-7^T^ and HSL3-1^T^ were 2.28 Mb and 2.49 Mb, respectively. The G+C contents of the genomic DNA of strains HSL1-7^T^ and HSL3-1^T^ were determined to be 36.1% and 57.3%, respectively (Table 1). The pairwise ANI values between HSL1-7^T^ and the closest related type strain *S. marina* B2^T^ was 89.2%, and the dDDH values between them were 36.9% (Table S1). The pairwise ANI values between strain HSL3-1^T^ and the closest related type strain *S. marina* B2^T^ were 67.41%, and the dDDH values between them were 23.7% (Table S2). All these values fell well below the established thresholds for prokaryotic species delineation (95–96% ANI and 70% dDDH) [51,52]. These data indicate that strains HSL1-7^T^ and HSL3-1^T^ represent two novel species of the genus *Sulfurimonas*.

**Figure 2:**
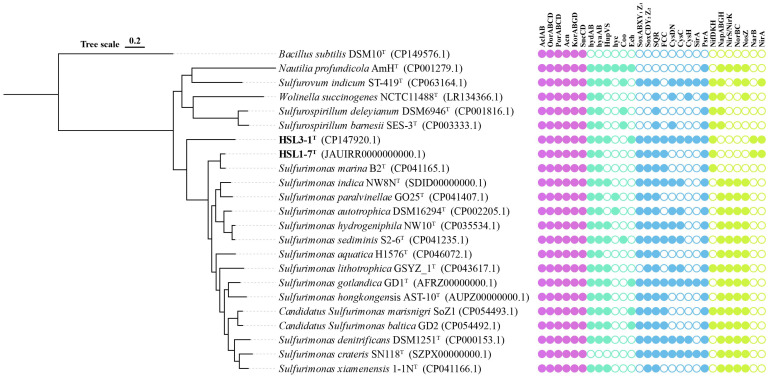
Phylogenetic reconstruction using 92 core genes was performed for strains HSL1-7^T^ and HSL3-1^T^, along with their functional genes involved in carbon, hydrogen, sulfur, and nitrogen metabolism, in comparison with closely related species. Bootstrap values (>70%) are indicated at nodes based on 1000 replicates. Species names highlighted in bold represent novel taxa discovered in this work. Bar, 0.2 substitutions per nucleotide position. Column descriptions are as follows: Carbon pathway: ATP citrate lyase alpha and beta subunits (AclAB), 2- oxoglutarate:ferredoxin oxidoreductase (OorABCD), and pyruvate:ferredoxin oxidoreductase (PorABCD), aconite hydratase (Acn), 2-oxoglutarate oxidoreductase alpha-, beta-, gamma-, and delta- subunits (KorABGD), succinyl-CoA synthetase alpha and beta subunits (SucCD). Hydrogen pathways: [NiFe]-Hydrogenases Group I (HydAB and HyaAB), [NiFe]-Hydrogenases Group II (HupVS), [NiFe]-Hydrogenases Group IV (Hyc, Coo and Ech). Sulfur pathways: thiosulfate oxidating Sox proteins (SoxABXY_1_Z_1_, SoxY_2_Z_2_CD), sulfide:quinone oxidoreductase (Sqr), flavocytochrome c sulfide dehydrogenase (Fcc), sulfate adenylyltransferase (CysDN), adenylylsulfate kinase (CysC), phosphoadenosine phosphosulfate reductase (CysH), ferredoxin-sulfite reductase (SirA), polysulfide reductase chain A (PsrA). Nitrogen pathways: nitrogenase molybdenum-iron (alpha- and beta-chains) and iron protein (NifDKH), periplasmic nitrate reductase components (NapABGH), nitrite reductase (NirK/NirS), nitric oxide reductase subunit A/B (NorBC), nitrous-oxide reductase (NosZ), ferredoxin-nitrate reductase (NarAB), ferredoxin-nitrite reductase (NirA).

The phylogenetic tree based on the 92 core gene sequences revealed that strain HSL1-7^T^ formed a distinct branch with *S. marina* B2^T^, while HSL3-1^T^ clustered together with other reference strains of *Sulfurimonas* (Figure 2). This result corroborated phylogenetic analyses of the 16S rRNA gene sequences, which supports that strains HSL1-7^T^ and HSL3-1^T^ represent members of the genus *Sulfurimonas*. Therefore, the genome-based phylogeny clearly indicates that strains HSL1-7^T^ and HSL3-1^T^ represent two novel species of the genus *Sulfurimonas*.

### 3.5. Genomic Functional Analysis

#### 3.5.1. Carbon Metabolism

The genome analysis revealed that strains HSL1-7^T^ and HSL3-1^T^ possess the complete set of enzymes required for carbon fixation via the reductive tricarboxylic acid (rTCA) cycle, including the key enzymes encoding ATP citrate lyase (AclAB), 2-oxoglutarate: ferredoxin oxidoreductase (OorABCD), and pyruvate:ferredoxin oxidoreductase (PorABCD) (Figure 2). This genome characteristic was accordant with the metabolic features of chemoautotrophic growth in strains HSL1-7^T^ and HSL3-1^T^, which was also conserved in the *Sulfurimonas*. In addition, the genomes of strains HSL1-7^T^ and HSL3-1^T^ contained enzymes for the intact tricarboxylic acid (TCA) cycle, including aconitehydratase (Acn), 2-oxoglutarateoxidoreductase (KorABGD), and succinyl-CoAsynthetase (SucCD) (Figure 2). Emerging evidence reveals the TCA cycle operates bidirectionally as a metabolic orchestrator, not only fueling oxidative phosphorylation but dynamically supplying core metabolic nodes (α-ketoglutarate, succinyl-CoA, and oxaloacetate) which coordinate anabolic flux partitioning through moonlighting enzyme-mediated substrate channeling [53].

#### 3.5.2. Hydrogen Metabolism

The genomic analysis revealed that strain HSL1-7^T^ contained one type of [Ni-Fe]-hydrogenase (Group I), while strain HSL3-1^T^ contained three types of [Ni-Fe]-hydrogenases (Group I, Group II, and Group IV) (Figure 2). Group I hydrogenases are membrane-bound enzymes that catalyze the respiratory oxidation of hydrogen, coupled to the reduction of electron acceptors [54]. Like most members of the genus *Sulfurimonas*, strains HSL1-7^T^ and HSL3-1^T^ both harbor two copies of Group I [Ni-Fe]-hydrogenase. This suggests that Group I hydrogenases might be essential for growth, like the genus *Sulfurimonas*. Group II hydrogenases are possibly involved in hydrogen sensing or energy conversion at low hydrogen concentrations [30]; this was only found in strain HSL3-1^T^ and some isolates from the seawater and sediments of the genus *Sulfurimonas*. Moreover, only strain HSL3-1^T^ contained Group IV hydrogenases Ech. However, neither strain HSL1-7^T^ nor HSL3-1^T^ contained Group IV hydrogenases Hyc and Coo (Figure 2).

#### 3.5.3. Sulfur Metabolism

The genomic analysis revealed that strains HSL1-7^T^ and HSL3-1^T^ harbor two distinct Sox gene clusters (*soxABXY_1_Z_1_* and *soxCDY_2_Z_2_*), which collectively constitute a complete Sox multienzyme system (Figure 2). While in the *Sulfurimonas*, only strains *Sulfurimonas xiamenensis* and *Sulfurimonas lithotrophica* had the *soxCDY_2_Z_2_* gene cluster [55]. Thus, the complete Sox gene clusters confer the capability of the isolated strains to oxidize thiosulfate to sulfate. Moreover, the genomes of strains HSL1-7^T^ and HSL3-1^T^ also contained flavocytochrome c sulfide dehydrogenase (Fcc) and sulfide:quinone oxidizing reductase (Sqr) for sulfide oxidation (Figure 2), which are also found in all members of genus *Sulfurimonas*. Only strain HSL3-1^T^ contained the complete enzymes required for the assimilatory sulfate reduction pathway, including the key enzymes sulfate adenylyltransferase (CysDN), adenylylsulfate kinase (CysC), phosphoadenosine phosphosulfate reductase (CysH), and ferredoxin-sulfite reductase (SirA), which are capable of reducing sulfate to organic sulfur compounds. Further, the genomes of strains HSL1-7^T^ and HSL3-1^T^ both encoded polysulfide reductase (Psr) (Figure 2), which could reduce elemental sulfur to sulfide.

#### 3.5.4. Nitrogen Metabolism

The comparative genome analysis revealed that strains HSL1-7^T^ and HSL3-1^T^ contain the complete nitrogen fixation pathway catalyzed by the nitrogenase encoded NifH (nitrogenase iron protein), NifD (nitrogenase Mo-iron alpha chain), and NifK (nitrogenase Mo-iron beta chain) (Figure 2). While in genus *Sulfurimonas*, only strains *S. marisnigri* SoZ1, *S. lithotrophica* GYSZ_1, *S. baltica* GD2, and *S. marina* B2 possessed the nitrogen fixation pathways [56]. Mangrove sediments are established as nitrogen-limited ecosystems due to the unbalance of carbon and nitrogen caused by the burying of plant litter or animal corpses. Nitrogen-fixing bacteria are considered to play a key role in nitrogen amendment [3]. Thus, the strains that contained nitrogenase genes in this study may have a competitive advantage in mangrove sediments. Moreover, strains HSL1-7^T^ and HSL3-1^T^ lack the genes encoding all the components required for the complete reduction of nitrate to nitrogen gas. Specifically, the genes for nitrate reductases (NapABGH), nitrite reductases (NirKS), nitric oxide reductases (NorBC), and nitrous oxide reductases (NosZ) were not detected in these strains (Figure 2), suggesting that they are unable to utilize nitrate as a terminal electron acceptor. In addition, strains HSL1-7^T^ and HSL3-1^T^ possessed the complete assimilatory nitrate reduction pathway, including the key enzyme encoding ferredoxin-nitrate reductase (NarAB) and ferredoxin-nitrite reductase (NirA).

### 3.6. Nitrogen Fixation Activity

**Figure 3:**
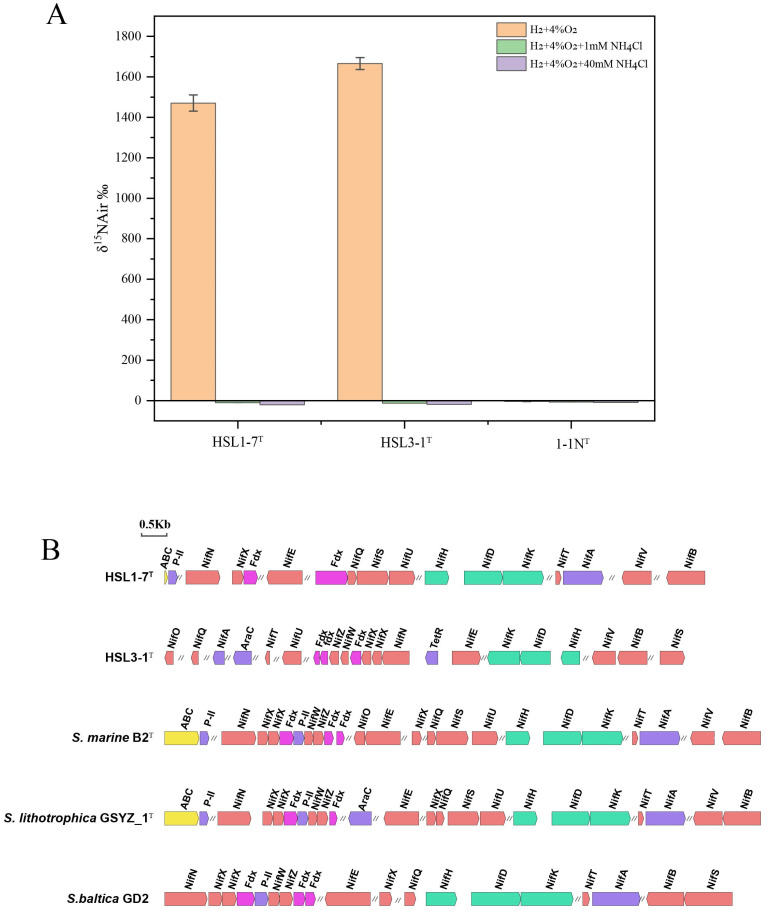
Determination of nitrogen fixation activity of strains HSL1-7^T^ and HSL3-1^T^ and analysis of nitrogen fixation gene clusters. (**A**) ^15^N abundance based on the ^15^N incorporation assay, the incubation conditions were H_2_ as the sole electron donor and O_2_ as the electron acceptor, *Sulfurimonas xiamenensis* 1-1N^T^ which lacked *nif* gene cluster served as a control. (**B**) Green represents nitrogen fixation genes (*nifHDK*), red represents nitrogenase metal cofactor biosynthesis genes (*nifBSZVENUWOQXTN*), purple represents transcriptional regulator genes (*P-II*, *nifA*, *araC*), pink represent electron transfer genes (*fdx*), and yellow represents transporter genes (*ABC*).

The ^15^N_2_ isotope labelling experiments demonstrated that both strains, HSL1-7^T^ and HSL3-1^T^, possessed the capability to fix ^15^N_2_ in the absence of an available nitrogen source in the medium, and that they exhibited similar nitrogen-fixing rates (Figure 3A). Whereas the reference strain *Sulfurimonas xiamenensis* 1-1N^T^ from the coastal sediments, which lacks nitrogen fixation gene clusters, could not perform nitrogen fixation. Moreover, ^15^N_2_ fixation in both strains were completely inhibited by the addition of ammonia, regardless of whether the concentration was low or high (1 mM and 40 mM of NH_4_Cl) (Figure 3A). This observation aligns with the fact that nitrogen fixation is often repressed when exogenous nitrogen sources are readily available in their environment [57]. While in *Sulfurimonas*, only strains *S. marina* B2^T^, *S. lithotrophica* GYSZ_1^T^, and *S. baltica* GD2 harbored the complete nitrogenase gene clusters, suggesting that they have the potential for nitrogen fixation (Figure 3B), but this has not been verified so far. These results suggest an important role for nitrogen fixation of these isolates in the nitrogen-limited mangrove ecosystems in which they may be present.

### 3.7. Taxonomic Conclusion

Based on these results, strains HSL1-7^T^ and HSL3-1^T^ represent two novel species of the genus *Sulfurimonas*, for which the names *Sulfurimonas microaerophilic* sp. nov. and *Sulfurimonas diazotrophicus* sp. nov. are proposed.

#### 3.7.1. Description of *Sulfurimonas microaerophilic* sp. nov.

*Sulfurimonas microaerophilic* (mi.cro.ae.ro’phi.lus. Gr. adj. mikros small, little; Gr. n. aer-aeros air; N.L. masc. adj. philus (from Gr. masc. adj. philos) loving; N.L. masc. adj. microaerophilus low-air loving, where the type strain was isolated).

The bacterial cells are Gram-stain-negative, rod-shaped to slightly curved (about 1.8–2.3 µm length × 0.4–0.6 µm in width), exhibiting motility via a single polar flagellum. The isolate was an obligate chemolithoautotroph capable of growing with molecular hydrogen and thiosulfate as an energy source, and molecular oxygen and elemental sulfur as the electron acceptors. Sodium bicarbonate and carbon dioxide can be used as inorganic carbon sources, whereas the growth of the strain was not observed under organic carbon source conditions such as peptone, yeast paste, and starch. The strain exhibited facultative microaerophilic physiology (growth up to 0–10% O_2_ in the gas phase; optimum growth in the presence of 4% O_2_). Growth parameters were established with temperature optima at 32 °C (range 4–40 °C), a salinity preference at 3.0% NaCl (*w*/*v*) (range 1.0–4.0%), and a pH optima at 7.0 (range 5.0–8.5). The major cellular fatty acids are C_16:0_ (31.6%), C_16:1_*ω7c* (28.7%), C_18:1_*ω7c* (19.5%), and C_14:0_ (10.5%).

The type strain, HSL1-7^T^ (=MCCC 1A18899^T^ = KCTC 25640^T^), was isolated from mangrove sediments of the Jiulong River tributaries in Zhangzhou, Fujian province, China. The genomic DNA G+C content of the type strain is 36.1%. The GenBank accession number for the 16S rRNA gene sequence is OQ102629, and the genome accession number is PRJNA989520, respectively.

#### 3.7.2. Description of *Sulfurimonas diazotrophicus* sp. nov.

*Sulfurimonas diazotrophicus* (di.a.zo.trophi.cus. Gr. pref. di two, double; N. L. n. azotum nitrogen; Gr. adj. trophikos tending or feeding; N. L. masc. adj. diazotrophicus, one that feeds on dinitrogen).

The bacterial cells are Gram-stain-negative, displaying rod-shaped morphology (approximately 0.7–2.5 µm length × 0.2–0.5 µm in width) with non-motile characteristics. As a strict chemolithoautotroph, the isolate utilizes molecular hydrogen and thiosulfate as an energy source, while employing molecular oxygen and elemental sulfur as terminal electron acceptors. Nitrogen assimilation occurs through ammonium ions, sodium nitrate, and dinitrogen fixation, whereas growth showed no requirement for selenium, tungsten, or vitamin supplementation. Nitrogen fixation is completely inhibited by NH_4_Cl. Growth occurs at 4–45 °C with optimum growth at 37 °C, and at pH 5.0–8.5 with an optimum at pH 7.0. Growth is observed in the presence of 2.0–4.0% (*w*/*v*) NaCl with an optimum at 3.0% NaCl (*w*/*v*). The major cellular fatty acids are C_16:1_*ω7c* (35.0%), C_16:0_ (29.6%), C_18:1_*ω7c* (16.7%), and C_14:0_ (11.3%).

The type strain, HSL3-1^T^ (=MCCC 1A18844^T^), was isolated from mangrove sediments of the Jiulong River tributaries in Zhangzhou, Fujian province, China. The G+C content of DNA was 57.3%. The GenBank accession number for the 16S rRNA gene sequence is OQ102631 and the genome accession number is PRJNA1085846, respectively.

## 4. Conclusions

The phylogenetic analysis based on the 16S rRNA gene sequences and whole genome, revealed that strains HSL1-7^T^ and HSL3-1^T^ represent two novel members of the genus *Sulfurimonas*. The 16S rRNA gene sequence similarity, and the ANI and dDDH values of strains HSL1-7^T^ and HSL3-1^T^ with closely related species, were lower than the thresholds for the delineation of a novel species. These results indicate that strains HSL1-7^T^ and HSL3-1^T^ do not represent any previously described species and should be considered to represent two novel species.

In addition, the physiological and biochemical characteristics of strains HSL1-7^T^ and HSL3-1^T^ differed from those of other species of the genus *Sulfurimonas* in terms of morphology, major fatty acid fractions, and patterns of electron donor-acceptor utilization. The comparative genomic analysis revealed that strains HSL1-7^T^ and HSL3-1^T^ possessed diverse metabolic profiles, including carbon fixation, hydrogen oxidation, sulfur oxidation, and nitrogen fixation. These unique metabolic capabilities suggests their potential role in driving the biogeochemical cycling of carbon, nitrogen, and sulfur in mangrove ecosystems. Notably, unlike most members of the genus *Sulfurimonas*, neither strain HSL1-7^T^ or HSL3-1^T^ genomes contained a complete denitrification pathway, but both harbored key genes for nitrogen fixation (*nifHDK*). Further, the ^15^N_2_ isotope labeling experiments demonstrated that they both exhibited strong nitrogen-fixing abilities. This characteristic implies that they may play an important role in the dark nitrogen fixation process in the mangrove ecosystem. The combined phenotypic, chemotaxonomic and phylogenetic evidence indicates that strains HSL1-7^T^ and HSL3-1^T^ represents two novel species in the genus *Sulfurimonas*. The findings of our study expand the understanding of *Sulfurimonas* species and their adaptation to mangrove environments.

## Figures and Tables

**Table 1:** Comparison of phenotypic characteristics of *Sulfurimonas microaerophilic* HSL1-7^T^ sp. nov. and *Sulfurimonas diazotrophicus* HSL3-1^T^ sp. nov. with related type strains within the genus *Sulfurimonas*.

Characteristic	1	2	3	4	5	6	7
Shape	Rods to slightly curved	Rods	Rods	Rods to slightly curved	Rods to slightly curved	Rods	Rods
Motility	+	−	+	+	−	−	−
Doubling time under optimal (h)	ND	ND	3.6	8	12	12	ND
Temperature range (Optimal T) (°C)	10–40 (32)	4–45 (37)	10–45 (35)	4–45 (33)	10–45 (30)	ND	15–35 (30)
pH range (Optimal pH)	5.0–8.5 (7.0)	5.0–8.5 (7.0)	4.5–9.0 (7.0)	5.0–8.5 (6.5)	5.5–8.0 (7.0)	ND (7.0)	6.5–8.5 (7.0–7.5)
NaCl requirement	+	+	+	+	−	−	+
Maximum O_2_ concentration (%)	10	20	15	20	20	0.5	0
Electron donor	H_2_, S_2_O_3_^2−^	H_2_, S_2_O_3_^2−^	HS^−^, S^0^, S_2_O_3_^2−^	H_2_, S^0^, S_2_O_3_^2−^, HS^−^	H_2_, S^0^, S_2_O_3_^2−^, S_4_O_6_^2−^, HS^−^	S_2_O_3_^2−^, HS^−^	H_2_, S_2_O_3_^2−^, HS^−^
Organic electron donors	−	−	−	−	−	Formate, fumarate, yeast extract alcohol mix	−
Electron acceptor	S^0^, O_2_	S^0^, O_2_	O_2_	S^0^, NO_3_^−^, O_2_	S^0^, NO_3_^−^, O_2_	NO_3_^−^, O_2_, NO_2_^−^	NO_3_^−^
DNA G+C content (%)	36.1	57.3	36.0	33.2	34.5	36	34.9

+, positive; −, negative; ND, not determined. Strains: 1, HSL1-7^T^; 2, HSL3-1^T^; 3, *S*. *marina* B2^T^ [24]; 4, *S*. *lithotrophica* GYSZ_1^T^ [19]; 5, *S*. *xiamenensis* 1-1N^T^ [19]; 6, *S. denitrificans* DSM 1251^T^ [14]; 7, *S. hongkongensis* AST-10^T^ [17].

**Table 2:** Cellular fatty acids compositions (%) of *Sulfurimonas microaerophilic* HSL1-7^T^ sp. nov. and *Sulfurimonas diazotrophicus* HSL3-1^T^ sp. nov. with related type strains within the genus *Sulfurimonas*.

Fatty acid (%)	1	2	3	4	5	6	7
C_12:0_	0.3	4.0	1.3	2.6	-	-	-
C_14:0_	**10.5**	**11.3**	9.1	3.2	1.1	0.4	4.8
C_14:0_ 3-OH	2.4	4.0	6.6	4.9	-	-	-
C_16:0_	**31.6**	**29.6**	**25.6**	**18.9**	**23.4**	**15.3**	**32.8**
C_16:1_*ω7c*	**28.7**	**35.0**	**37.7**	**50.5**	**31.8**	**67.9**	-
C_16:1_*ω5c*	0.6	0.4	-	-	-	2.0	-
C_18:0_	2.6	0.7	1.1	1.3	**10.0**	-	**16.9**
C_18:1_*ω*7*c*	**19.5**	**16.7**	**13.2**	**12.1**	**18.7**	**12.1**	-

Fatty acids present at levels exceeding 10% were highlighted by bold type. -, not detected. Strains: 1, HSL1-7^T^; 2, HSL3-1^T^; 3, *S. marina* B2^T^; 4, *S. lithotrophica* GYSZ_1^T^; 5, *S. xiamenensis* 1-1N^T^; 6, *S. denitrificans* DSM 1251^T^; 7, *S. hongkongensis* AST-10^T^.

## Data Availability

The GenBank accession number for the 16S rRNA gene sequence and complete genome sequence of *Sulfurimonas microaerophilic* HSL1-7^T^ are OQ102629 and PRJNA989520, respectively. The GenBank accession number for the 16S rRNA gene sequence and complete genome sequence of *Sulfurimonas diazotrophicus* HSL3-1^T^ are OQ102631 and PRJNA1085846, respectively. Sulfurimonas microaerophilic HSL1-7: The 16S rRNA gene sequence can be accessed in GenBank under accession number OQ102629 via the following link: https://www.ncbi.nlm.nih.gov/search/all/?term=OQ102629 accessed on 20 October 2024; The complete genome sequence can be accessed in GenBank under accession number PRJNA989520 via the following link: https://www.ncbi.nlm.nih.gov/search/all/?term=PRJNA989520 accessed on 20 October 2024; Sulfurimonas diazotrophicus HSL3-1: The 16S rRNA gene sequence can be accessed in GenBank under accession number OQ102631 via the following link: https://www.ncbi.nlm.nih.gov/search/all/?term=OQ102631 accessed on 20 October 2024; The complete genome sequence can be accessed in GenBank under accession number PRJNA1085846 via the following link: https://www.ncbi.nlm.nih.gov/search/all/?term=PRJNA1085846 accessed on 20 October 2024.

## Supplementary Materials

The following supporting information can be downloaded at: https://www.mdpi.com/article/10.3390/microorganisms13040713/s1, Figure S1: Transmission electron micrographs of cells of *Sulfurimonas microaerophilic* HSL1-7^T^ (a) and *Sulfurimonas diazotrophicus* HSL3-1^T^ (b); Figure S2: Neighbor joining phylogenetic tree based on 16S rRNA gene sequences showing the relationship between strains HSL1-7^T^ and HSL3-1^T^ with other members within the genus *Sulfurimonas*; Figure S3: Minimum-evolution phylogenetic tree based on 16S rRNA gene sequences showing the relationship between strains HSL1-7^T^ and HSL3-1^T^ with other members within the genus *Sulfurimonas*; Table S1: The average nucleotide identity (ANI) and in silico DNA-DNA hybridization values (dDDH) between strain HSL1-7^T^ and related strains of the genus *Sulfurimonas*; Table S2: The average nucleotide identity (ANI) and in silico DNA-DNA hybridization values (dDDH) between strain HSL3-1^T^ and related strains of the genus *Sulfurimonas*.
