# Supplementary material for: Sulfurimonas microaerophilic sp. nov. and Sulfurimonas diazotrophicus sp. nov.: Two Novel Nitrogen-Fixing and Hydrogen- and Sulfur-Oxidizing Chemolithoautotrophs Within the Campylobacteria Isolated from Mangrove Sediments
Source: Microorganisms. 2025 Mar 21;13(4):713. doi: 10.3390/microorganisms13040713 (PMC12029903; doi:10.3390/microorganisms13040713)
Supplement: Supplementary file 1 [file microorganisms-13-00713-s001.zip › KCTC 25640.pdf]

Korean Collection for Type Cultures (KCTC)
Korea Research Institute of Bioscience and Biotechnology (KRIBB)
181 Ipsin-gil, Jeongeup-si, Jeonbuk 56212, South Korea
Tel: +82-63-570-5602, FAX: +82-63-570-5609
E-mail: deposit@kribb.re.kr

Certificate of Deposit

Ref.: 20230738 Date of issue: MAY 11, 2023

Taxonomic designation	:	***Sulfurimonas sp.***
Accession number	:	**KCTC 25640**
Depositor(s)	:	Zongze Shao
Strain code by the depositor(s)	:	**HSL1-7**

The above microorganism has been successfully deposited into the general collection of microorganism of the Korean Collection for Type Cultures (KCTC) and confirmed the identity of the microorganism under this KCTC number.
This microorganism will be available without restrictions for research and academic purposes in the publicly accessible section of the KCTC. It will be included in published and online catalogues after publication of this number by the authors.

Curator of Bacteria
Jung-Sook Lee Ph.D.

Telephone: +82-63-570-5618
Fax: +82-63-570-5609
E-mail: jslee@kribb.re.kr
Web: http://kctc.kribb.re.kr
