# Supplementary material for: Sulfurimonas microaerophilic sp. nov. and Sulfurimonas diazotrophicus sp. nov.: Two Novel Nitrogen-Fixing and Hydrogen- and Sulfur-Oxidizing Chemolithoautotrophs Within the Campylobacteria Isolated from Mangrove Sediments
Source: Microorganisms. 2025 Mar 21;13(4):713. doi: 10.3390/microorganisms13040713 (PMC12029903; doi:10.3390/microorganisms13040713)
Supplement: Supplementary file 1 [file microorganisms-13-00713-s001.zip › MCCC 1A18844.pdf]

中国海洋微生物菌种保藏管理中心
Marine Culture Collection of China

CERTIFICATE OF DEPOSIT

IN MARINE CULTURE COLLECTION OF CHINA

Marine Culture Collection of China
Third Institute of Oceanography, Ministry of Natural Resources
No. 178 Daxue Road, 361005 Xiamen, Fujian Province
P. R. China.
Phone/Fax: +86-592-2195177
Email: mccc5177@163.com
Web site: http://www.mccc.org.cn

MCCC 1A18844

***Sulfurimonas* sp. (strain HSL3-1) was received for deposit**
in Marine Culture Collection of China from

Lijing Jiang

Third Institute of Oceanography, State Oceanic Administration
Daxue Road 178, Xiamen 361005, Fujian
P. R. China

on Oct. 27, 2021

and was, after confirming the viability and purity,
allocated the accession number MCCC 1A18844.

The strain is available to any *bona fide* scientific community or individual,
operating in a professional environment
suitable for handling living material of the biohazard group involved.

Xiamen, Nov. 2, 2021

Dr. Zongze Shao
Public Collection Curator
Marine Culture Collection of China
