# Supplementary material for: Sulfurimonas microaerophilic sp. nov. and Sulfurimonas diazotrophicus sp. nov.: Two Novel Nitrogen-Fixing and Hydrogen- and Sulfur-Oxidizing Chemolithoautotrophs Within the Campylobacteria Isolated from Mangrove Sediments
Source: Microorganisms. 2025 Mar 21;13(4):713. doi: 10.3390/microorganisms13040713 (PMC12029903; doi:10.3390/microorganisms13040713)
Supplement: Supplementary file 1 [file microorganisms-13-00713-s001.zip › MCCC 1A18899.pdf]

国家菌种资源库
NMRC National Microbial Resource Center
MCCC org.cn
中国海洋微生物菌种保藏管理中心
Marine Culture Collection of China

# CERTIFICATE OF DEPOSIT

# IN MARINE CULTURE COLLECTION OF CHINA


**Marine Culture Collection of China**
**Third Institute of Oceanography, Ministry of Natural Resources**
**No. 178 Daxue Road, 361005 Xiamen, Fujian Province**
**P. R. China.**
Phone/Fax: +86-592-2195177
Email: mccc5177@163.com
Web site: http://www.mccc.org.cn


## MCCC 1A18899

***Sulfurimonas* sp. (strain HSL1-7) was received for deposit**
**in Marine Culture Collection of China from**

**Lijing Jiang**

**Third Institute of Oceanography, State Oceanic Administration**
**Daxue Road 178, Xiamen 361005, Fujian**
**P. R. China**

**on Dec. 6, 2021**

**and was, after confirming the viability and purity,**
**allocated the accession number MCCC 1A18899.**

**The strain is available to any *bona fide* scientific community or individual,**
**operating in a professional environment**
**suitable for handling living material of the biohazard group involved.**

**Xiamen, Jan. 10, 2022**

**Dr. Zongze Shao**
**Public Collection Curator**
**Marine Culture Collection of China**
