# Supplementary material for: Sulfurimonas microaerophilic sp. nov. and Sulfurimonas diazotrophicus sp. nov.: Two Novel Nitrogen-Fixing and Hydrogen- and Sulfur-Oxidizing Chemolithoautotrophs Within the Campylobacteria Isolated from Mangrove Sediments
Source: Microorganisms. 2025 Mar 21;13(4):713. doi: 10.3390/microorganisms13040713 (PMC12029903; doi:10.3390/microorganisms13040713)
Supplement: Supplementary file 1 [file microorganisms-13-00713-s001.zip › Supplementary_Materials.pdf]

Supplementary Materials

Sulfurimonas diazotrophicus sp. nov., and *Sulfurimonas microaerophilic* sp. nov., two novel nitrogen-fixing and hydrogen- and sulfur-oxidizing chemolithoautotrophs within the *Campylobacteria* isolated from mangrove sediments

Yangsheng Zhong[1,2,3#], Yufei Li[1,2,3#], Zhaodi Wang[1,2,3], Liang Cui[1,2,3], Shiwei lv[1,2,3], Han Zhu[1,2,3], Qing Yuan[1,2,3], Qiliang Lai[1,2,3], Shasha Wang[1,2,3]* and Lijing Jiang[1,2,3]*

[1] Key Laboratory of Marine Genetic Resources, Third Institute of Oceanography, Ministry of Natural Resources of PR China, Xiamen 361005, PR China

[2] State Key Laboratory Breeding Base of Marine Genetic Resources, Third Institute of Oceanography, Ministry of Natural Resources of PR China, Xiamen 361005, PR China

* Correspondence: Shasha Wang (wangshasha@tio.org.cn); Lijing Jiang (Jianglijing@tio.org.cn)

Keywords: *Sulfurimonas diazotrophicus* HSL1-7^T; *Sulfurimonas microaerophilus* HSL3-1^T; taxonomy; nitrogen fixation; sulfur oxidation

Supplementary Materials

Figure S1. a, Transmission electron micrograph of cells of *Sulfurimonas diazotrophicus* HSL1-7^T; b, Transmission electron micrograph of cells of *Sulfurimonas microaerophilic* HSL3-1^T.

Figure S2. Neighbor joining phylogenetic tree based on 16S rRNA gene sequences showing the relationship between strains HSL1-7^T and HSL3-1^T with other members within the genus *Sulfurimonas*.

Figure S3. Minimum-evolution phylogenetic tree based on 16S rRNA gene sequences showing the relationship between strains HSL1-7^T and HSL3-1^{T}with other members within the genus *Sulfurimonas*.

Table S1. The average nucleotide identity (ANI) and digital DNA-DNA hybridization values (dDDH) between strains HSL1-7^T and related strains of the genus *Sulfurimonas*.

Table S2. The average nucleotide identity (ANI) and digital DNA-DNA hybridization values (dDDH) between strains HSL3-1^T and related strains of the genus *Sulfurimonas*.

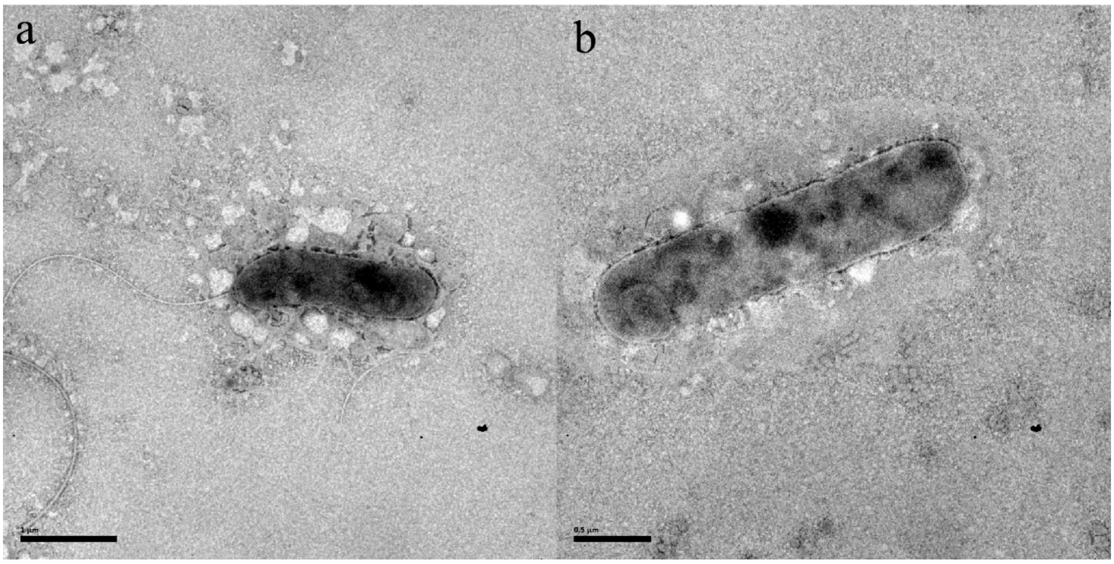

Figure S1. Transmission electron micrograph of cells of *Sulfurimonas diazotrophicus* HSL1-7^{T}(a) and *Sulfurimonas microaerophilic* HSL3-1^{T} (b). Bars, 1 μm (a)/0.5 μm (b).

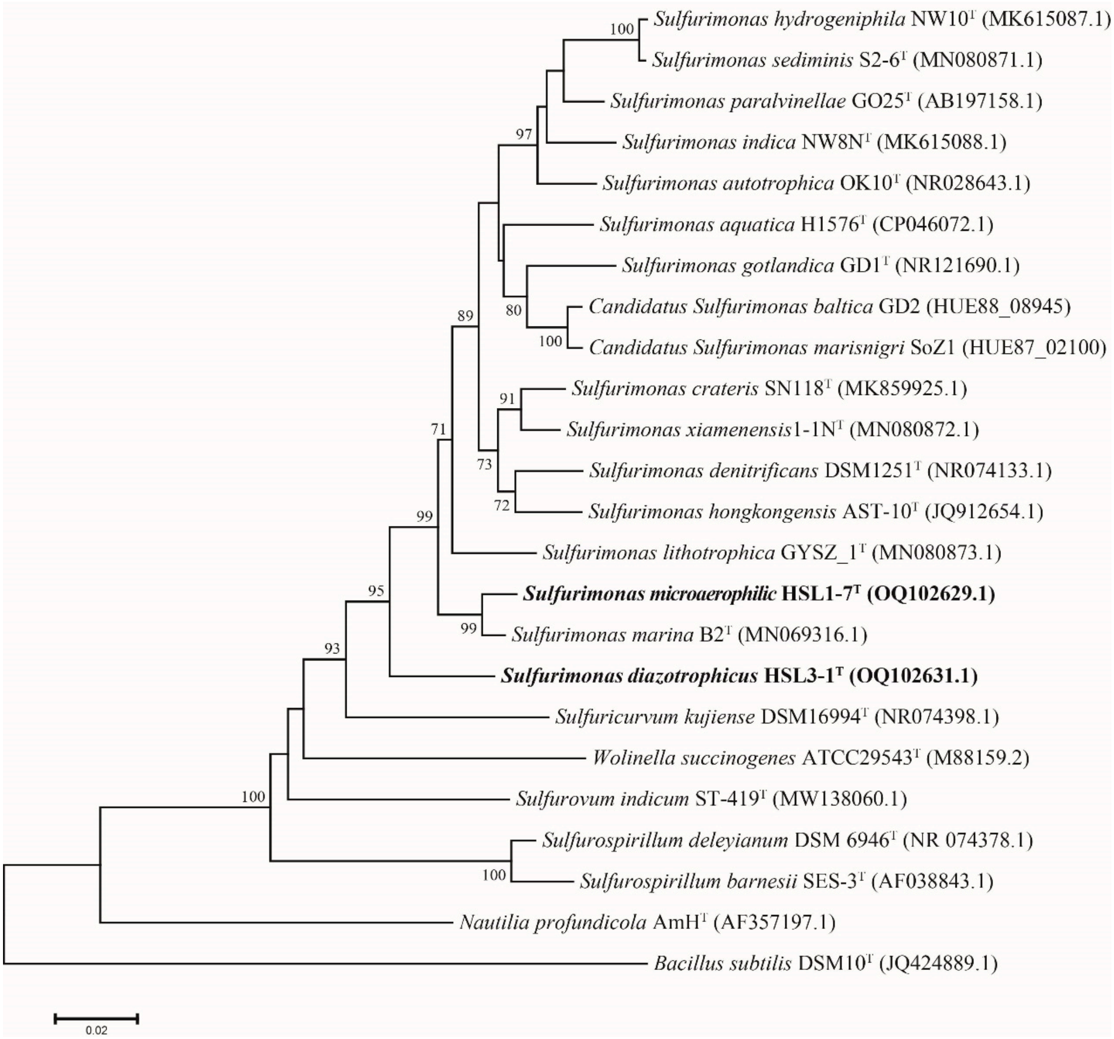

Figure S2. Neighbor joining phylogenetic tree based on 16S rRNA gene sequences showing the relationship between strains HSL1-7^T and HSL3-1^T with other members within the genus *Sulfurimonas*. Bootstrap values based on 1000 replicates (> 70%) are shown at branch nodes. The bold font represents the novel species identified in this study. Bacillus subtilis DSM10 (JQ424889) as the out group. Bar, 0.02 substitutions per nucleotide position.

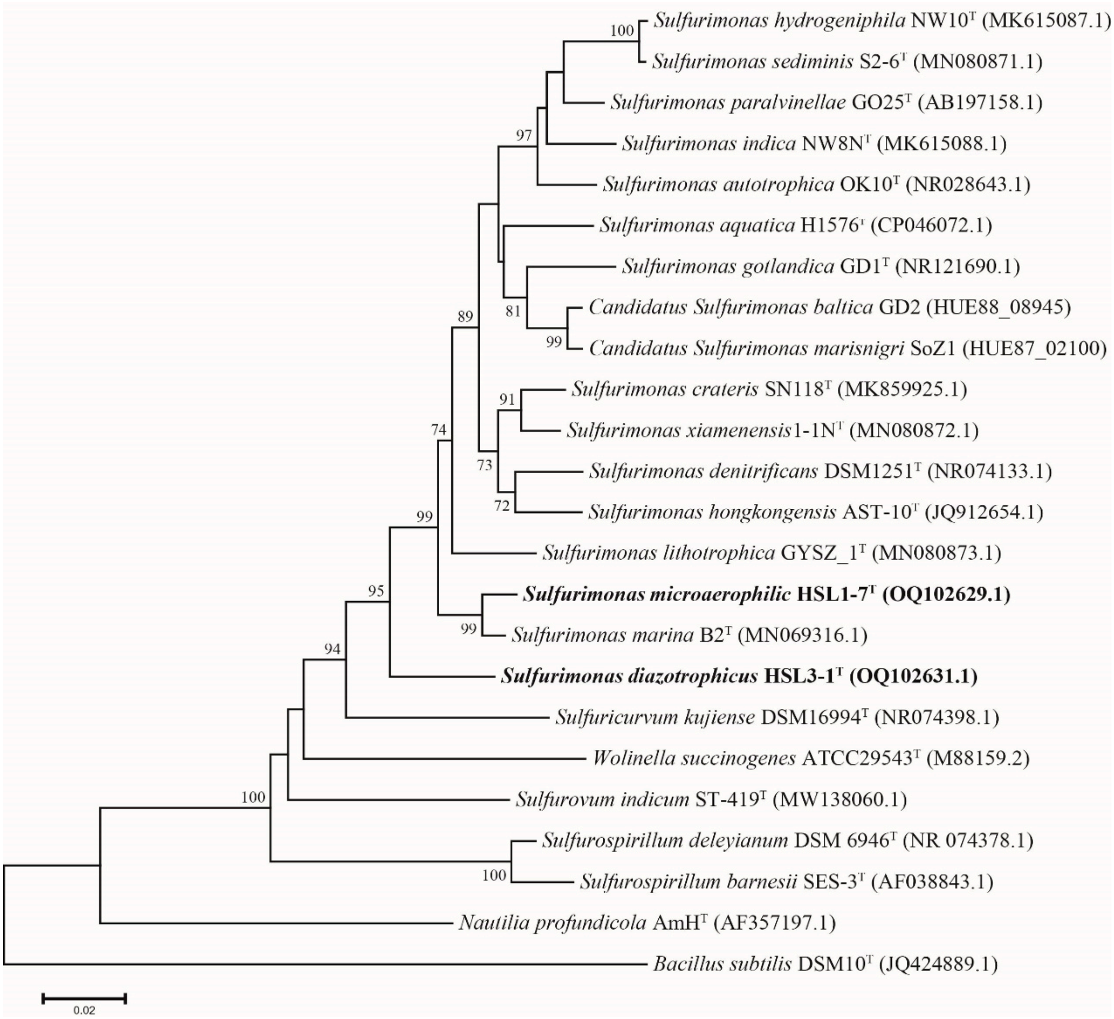

Figure S3. Minimum-evolution phylogenetic tree based on 16S rRNA gene sequences showing the relationship between strains HSL1-7^{T} and HSL3-1^{T}with other members within the genus *Sulfurimonas*. Bootstrap values based on 1000 replicates (> 70%) are shown at branch nodes. The bold font represents the novel species identified in this study. Bacillus subtilis DSM10 (JQ424889) as the out group. Bar, 0.02 substitutions per nucleotide position.

Table S1. The average nucleotide identity (ANI) and digital DNA-DNA hybridization values (dDDH) between strains HSL1-7^T and related strains of the genus *Sulfurimonas*.

	S. marina B2^T	*S. lithotrophica* GSYZ_1^T	*S. xiamenensis* 1-1N^T	*S. gotlandica* GD1^T
ANI (%)	89.2	72.4	71.5	72.04
dDDH(%)	36.9	19.4	19.4	19.3

Table S2. The average nucleotide identity (ANI) and digital DNA-DNA hybridization values (dDDH) between strains HSL3-1^{T} and related strains of the genus *Sulfurimonas*.

	S. marina B2^{T}	*S. lithotrophica* GSYZ_1^{T}	*S. crateris* SN118^{T}	*S. xiamenensis* 1-1N^{T}
ANI (%)	67.4	67.0	67.0	67.2
dDDH(%)	23.7	26.1	26.1	32.4
